# A Multimodal Framework to Guide Solid Tumor Indications for Natural Killer Cell‐Based Therapy

**DOI:** 10.1002/eji.70283

**Published:** 2026-09-16

**Authors:** L. Chiossone, V. Picant, Q. Barbier, T. A. Fehniger, Eric Vivier

**Affiliations:** ^1^ Innate Pharma Marseille France; ^2^ Washington University School of Medicine St. Louis Missouri USA; ^3^ Aix Marseille Université CNRS, INSERM, Centre d'Immunologie de Marseille‐Luminy Marseille France; ^4^ APHM, Hôpital de La Timone, MIPP Marseille France; ^5^ Paris Saclay Cancer Cluster Villejuif France; ^6^ Ecole Polytechnique Palaiseau France

**Keywords:** cancer research, clinical trial, immunotherapy, medicine, natural killer cell, transcriptome, tumor microenvironment

## Abstract

T cell‐based immunotherapies have achieved remarkable success in hematological malignancies but remain limited in solid tumors. Natural killer (NK) cells offer a compelling complementary approach, given their ability to recognize and kill tumor cells independently of neoantigen presentation and MHC class I expression. This study integrates multiomic analyses, clinical trial data, and transcriptomic analyses to systematically identify solid tumor indications most likely to benefit from NK cell‐based therapies. Immunohistochemical analysis revealed that NK cells infiltrate multiple tumor types, with the highest abundance in kidney tumors. Systematic review of 177 clinical trials identified variable efficacy across solid tumor indications, without a clearly superior tumor type or intervention. Transcriptomic profiling of over 10,000 TCGA samples assessed NK cell infiltration, cytotoxic fitness, and tumor microenvironment composition, highlighting kidney renal cell carcinoma, lung adenocarcinoma and squamous cell carcinoma, cervical and endocervical cancers, and mesothelioma as promising candidates for future clinical evaluation. Together, these findings provide a multimodal framework to prioritize tumor indications and improve the rational design of future NK cell clinical trials.

## Graphical abstract

This study integrates immunohistochemical evidence of NK cell infiltration in solid tumors, clinical outcomes of NK cell‐based therapies, and transcriptomic analyses of more than 10,000 TCGA samples. Together, these complementary datasets provide a multimodal framework for prioritizing tumor indications and informing the rational design of future NK cell‐based clinical trials.

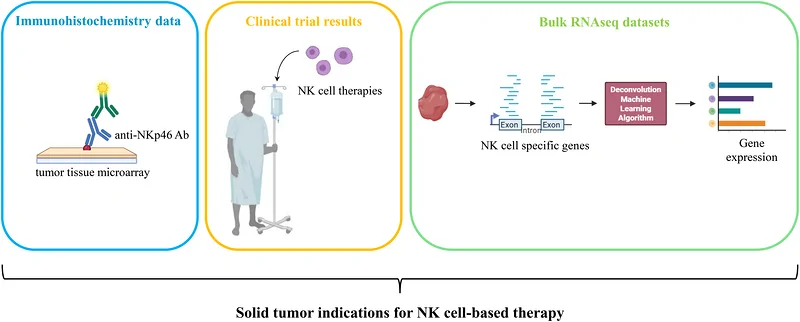

## 1 Introduction

T cell‐based immunotherapies have yielded undeniable clinical success in the treatment of hematological malignancies [1, 2, 3, 4] while much more modest results were observed in solid tumors, with only a handful of T cell‐based products recently approved by the FDA: lifileucel (autologous tumor‐infiltrating lymphocytes) and afami‐cel (TCR‐engineered autologous T cells) in advanced melanoma and synovial sarcoma [5, 6], respectively, as well as the T cell‐engaging bispecifics tebentafusp (anti‐gp100/HLA‐A*02:01 × CD3) in uveal melanoma [7] and tarlatamab (anti‐DLL3 × CD3) in extensive‐stage small cell lung cancer [8]. Moreover, the number of patients who experience durable responses is still limited, prompting the need for complementary treatment approaches. Natural killer (NK) cells play multifaceted roles in tumor control, namely direct cytotoxicity and the secretion of proinflammatory cytokines. Moreover, they recognize stressed cells independently of neoantigen presentation and exhibit increased cytotoxicity toward tumors that acquire resistance to T cell immunity via loss of MHC class I expression [9, 10, 11].

NK cells were evaluated as a cellular therapy for blood cancers after the discovery that NK cell alloreactivity predicted progression‐free survival for acute myeloid leukemia (AML) patients in the setting of MHC‐haploidentical hematopoietic cell transplant (HCT) [12]. In the first phase 1 clinical trial of allogeneic NK cells, Miller and colleagues utilized enriched conventional NK (cNK) cell products from MHC‐haploidentical donors following lymphodepleting chemotherapy and support with rhIL‐2 [13]. They showed that ∼25% of rel/ref AML patients safely achieved a composite complete remission (CR) without the need for an HCT, albeit durable for only a few months. Expansions of this approach incorporated Treg depletion; altered IL‐2 schedules also safely induced remissions at a higher rate than without Treg depletion (53% vs. 21%), and 33% of patients had ongoing remissions at 6 months [14]. Notably, NK cell expansion in the blood was significantly associated with clinical response. In a complementary approach, a phase 1 clinical trial utilized IL‐12, IL‐15, and IL‐18 pre‐activation to induce memory‐like programs prior to adoptive transfer of purified donor NK cells [15]. This resulted in a composite CR rate of 47% in rel/ref AML patients, with durations of remission lasting 6–9 months. Numerous trials of allogeneic nonengineered blood NK cells have shown improvements in CR rate and/or PFS compared with historical controls in early‐phase clinical trials, highlighting the promise of NK cell therapies [16, 17], with a consensus in the field that nonengineered NK cells can result in clinical benefits to patients with blood cancers, especially AML. However, large prospective randomized trials required for broad adoption of allogeneic NK cell therapy have not been performed in AML to support their broad use in AML and related diseases. This is most likely due to the challenges of commercialization and the advent of NK cell engineering strategies that provide new approaches to enhance NK cell responses to cancer.

With the success of cellular engineering to introduce CAR into NK cells [18], most clinical trials utilizing NK cells are modifying NK cells to express synthetic activating receptors, thereby providing additional activating signals and, in some cases, cytokine support [19]. The first CD19‐CAR‐engineered cord blood‐derived NK cells tested in the clinic had a promising early CR rate [20], and demonstrated that allogeneic CD19‐CAR NK cells could produce durable CRs in lymphoma patients with a 12‐month PFS of 32% [21]. Similarly, CD19‐CAR NK cells differentiated from iPSCs demonstrated clinical activity in a heterogeneous set of B cell lymphoma patients, including those that had progressed after autologous CAR T cells [22]. Based on these early successes, numerous ongoing clinical trials are exploring a variety of CAR targets and strategies to enhance the engineered NK cell activity, including targeting solid tumors [23, 24]. However, the effectiveness of NK cell‐based therapies in solid tumors remains inadequate, largely due to an incomplete understanding of tumor‐infiltrating NK cells, especially the mechanisms governing their infiltration or exclusion, their phenotypic and functional heterogeneity, and their functional dysregulation within the tumor microenvironment.

Given the pronounced heterogeneity of solid tumors, both in immune cell infiltration and in tumor cell intrinsic features such as the expression of activating or inhibitory NK cell ligands and the secretion of chemokines that regulate NK cell recruitment [25, 26], we aimed to identify solid tumor types most likely to benefit from therapies that potentiate NK cell activity. Focusing on these solid tumor indications will refine the translational relevance of NK cell‐based strategies and enhance the probability of achieving meaningful clinical benefit. Our strategy integrates large‐scale genomic dataset analyses with insights derived from immunohistochemistry and clinical trials evaluating NK cell infusions, enabling a comprehensive understanding of factors influencing therapeutic efficacy.

## 2 NK Cells Infiltrate Multiple Solid Tumor Types and Are Enriched in Renal Tumors

**FIGURE 1:**
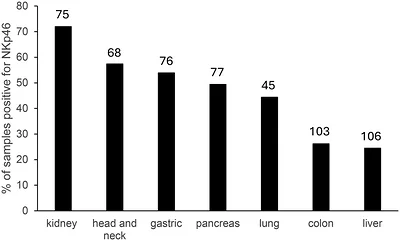
NK cell distribution across solid tumor indications. Commercially available formalin‐fixed paraffin‐embedded (FFPE) tumor tissue microarrays (lung (#LC10012a), pancreas (#PA1001b), gastric (#ST1003a), kidney (#HKid‐CRCC150CS‐02), colon (#BC051110b), head and neck (#HN802b) and liver (#BC03119a) from US Biomax) were stained with an anti‐NKp46 Ab (clone 8E5B, Innate Pharma) and anti‐mouse/rabbit. IgG detection system (Ultraview universal DAB detection kit, Ventana) was used for primary Ab detection and amplification [28]. Black bars represent the percentage of tumor samples positive where at least one NKp46^+^ cell was detected; numbers represent the number of samples tested for each tumor indication.

NKp46 is the canonical NK cell marker used in tissue sections [27]. We analyzed immunohistochemistry data generated by Gautier et al. [28] reporting the presence of NK cells in samples from human solid tumors (lung, pancreas, gastric, kidney, colon, head and neck, and liver) using anti‐NKp46 staining to identify which indications are characterized by a greater abundance of NK cells (Figure 1). NK cells were detectable across all tumor indications analyzed, with the highest detection rate observed in kidney tumors.

## 3 Efficacy of NK Cell Therapy across Solid Tumor Types

**FIGURE 2:**
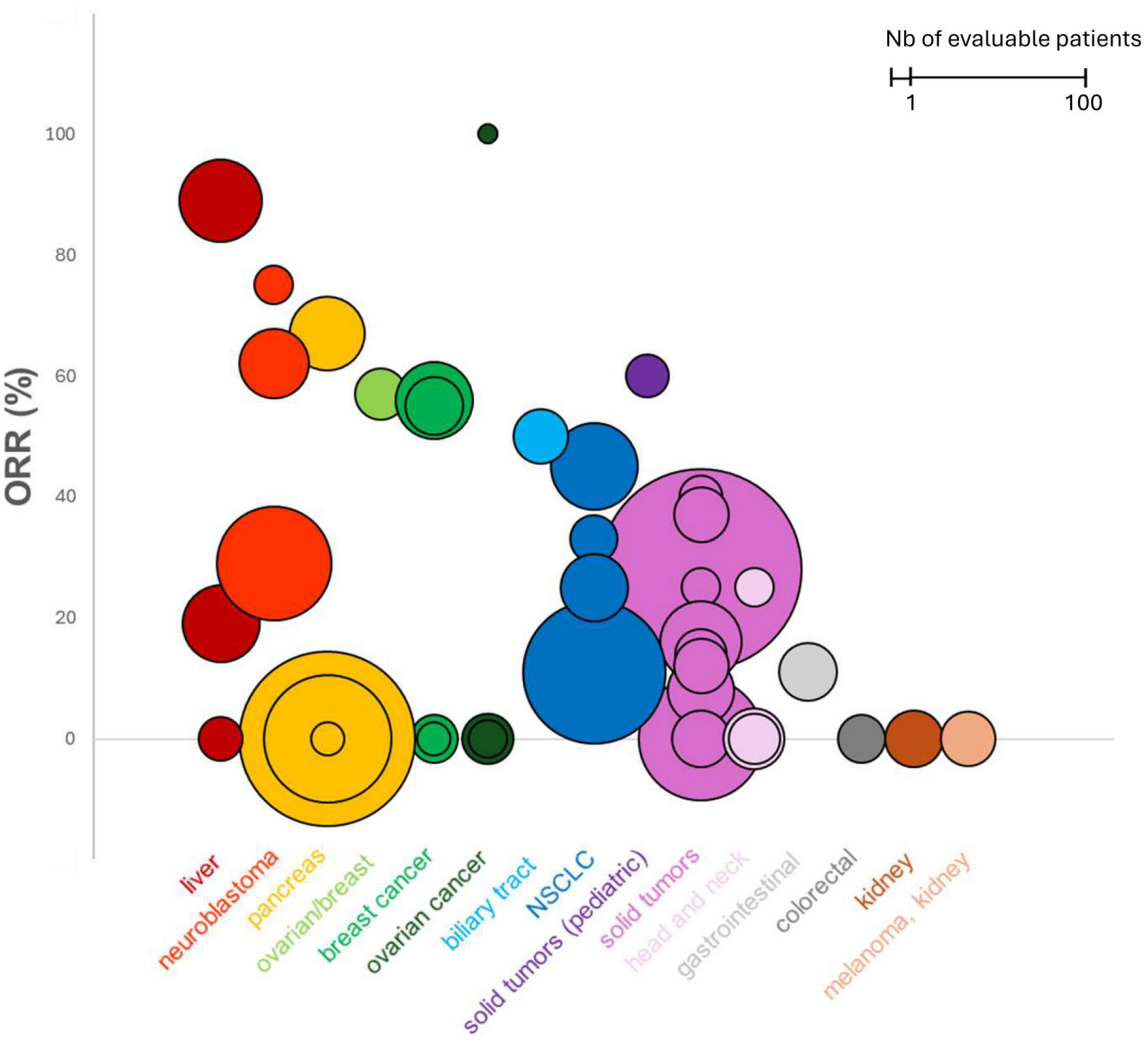
Clinical efficacy of NK cell infusions in patients with solid tumors. Clinical activity was assessed using the overall response rate (ORR), defined as the proportion of patients with complete or partial responses among all evaluable patients. Each bubble represents a clinical trial, and the size of the bubbles represents the number of evaluable patients.

To analyze in which solid tumors NK cell infusions showed the most significant clinical results, we conducted a search on the ClinicalTrials.gov database looking for all clinical studies involving NK cell infusions in solid tumors started from January first, 2000, to June 30, 2025. The search strategy applied the terms: condition‐tumor, intervention‐NK cells, status‐all studies. 807 trials were identified, of which 177 were retained after manual screening for relevance to solid tumors and the use of NK cells as an intervention. Among them, 83 clinical trials explored the use of NK cells as a single agent, while in the other 94 studies NK cells were used in combination with chemotherapy, pro‐inflammatory cytokines (mainly IL‐2 or IL‐15), or antibodies (targeting tumor antigens or immune checkpoint inhibitors). In nearly two‐thirds of the studies (114 out of 177), NK cells were not genetically modified (autologous or allogeneic), while 53 out of 177 trials used CAR‐NK cells and only 10 NK‐92 cell line. For 49 studies, efficacy results could be found on the Beacon database (data.beacon‐intelligence.com), and they are expressed as overall response rate (ORR) in Figure 2. Table S1 summarizes interventions for each clinical trial. The number of clinical trials reporting efficacy outcomes remains limited for each tumor indication. In addition, substantial variability in clinical responses was observed across studies, potentially reflecting differences in prior lines of therapy received before NK cell infusion. As a result, no single tumor indication or specific intervention could so far be clearly identified as demonstrating superior clinical benefit.

## 4 Clustering of Tumor Indications Based on Transcriptomic Data to Identify Functional NK Cell‐Enriched Tumors

**FIGURE 3:**
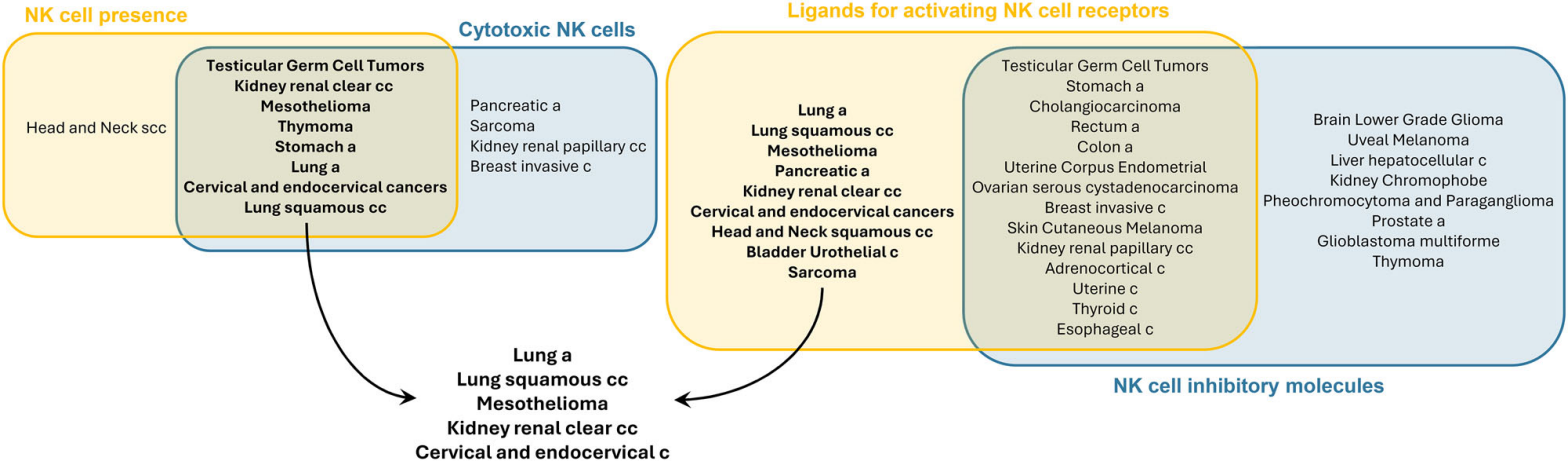
Solid tumor indication selection by clustering from transcriptomic dataset analysis. By heatmap analysis of RNA‐seq datasets from the TCGA database across 31 solid tumor types, assessing the expression of genes indicating NK cell presence and fitness, activating NK cell receptor ligands, and NK cell inhibitory molecules, a selection of tumor indications most likely to respond to NK cell‐based therapies was proposed. First, the expression of genes significantly overexpressed by NK cells (CHST12, KLRF1, GNLY, KLRC1, IL18RAP, KLRD1, IL2RB, CST7, XCL2, CD244, PRF1, NCR1, KIR2DL1, KIR2DL3, CD160, KLRC3) [29] and the expression of the activating NK cell receptors NKp30, CD16, KLRF1, CD160, DNAM1, KLRK1, CD244 and of the cytotoxic molecules perforin and granzyme B were analyzed to identify the presence of cytotoxic NK cells (left). Then, the presence of positive or negative NK cell regulators in the tumor microenvironment (ligands for activating and inhibitory NK cell receptors and soluble factors such as TGF‐β, PGE2, and IL‐10) was evaluated to detect tumors exhibiting microenvironmental features favorable to NK cell‐mediated lysis (right). Tumor types identified by both analyses are highlighted in bold. a, adenocarcinoma; cc, cell carcinoma; c, carcinoma.

To further explore the solid tumor conditions presenting the highest chances to respond to NK cell therapies, we analyzed the various RNA‐seq datasets available from the TCGA database of more than 10,000 tumor samples for 31 different types of cancer to assess NK‐cell infiltration, NK‐cell fitness, and microenvironmental factors (Table S2). Informative genes significantly overexpressed by NK cells (CHST12, KLRF1, GNLY, KLRC1, IL18RAP, KLRD1, IL2RB, CST7, XCL2, CD244, PRF1, NCR1, KIR2DL1, KIR2DL3, CD160, and KLRC3) [29] were selected to infer the abundance of these cell types in the tumor samples analyzed. By heatmap analysis, a group of tumors characterized by high NK cell infiltration emerged (Figure S1A). Then, the expression of the activating NK cell receptors NKp30, CD16, KLRF1, CD160, DNAM1, KLRK1, CD244 and of the cytotoxic molecules perforin and granzyme B was used to identify tumors containing NK cells with the highest cytotoxic potential [30] (Figure S1B). Within the tumor microenvironment, the balance of ligands for activating and inhibitory NK cell receptors, along with immunosuppressive soluble factors such as TGF‐β, PGE2, and IL‐10, shapes NK cell function [31, 32]. Analysis of the expression of these molecules (Figure S1C–D) delineated a subset of tumors exhibiting microenvironmental features favorable to NK cell‐mediated lysis, including kidney renal clear cell carcinoma, lung adenocarcinoma and squamous cell carcinoma, mesothelioma, pancreatic adenocarcinoma, cervical and endocervical cancers, head and neck squamous cell carcinoma, bladder urothelial carcinoma and sarcoma. Notably, lung, kidney, cervical and endocervical cancers and mesothelioma also displayed substantial infiltration by functional NK cells (Figure S1A–B). Collectively, these findings suggest that kidney renal cell carcinoma, lung adenocarcinoma and squamous cell carcinoma, cervical and endocervical cancers, and mesothelioma may be promising candidates for NK cell‐based therapies (Figure 3). However, it should be noted that these conclusions are based on bulk transcriptomic analyses, which are inherently limited by the heterogeneity of tumor samples, in terms of immune cell infiltration, tumor stage, and prior treatments.

Indications characterized by high intratumoral NK‐cell infiltration and fitness may be particularly suitable for therapeutic strategies leveraging endogenous NK cells in vivo (e.g., antibodies or immunocytokines). In contrast, indications displaying favorable expression patterns of activating ligands together with limited inhibitory signals may represent promising settings for adoptive NK‐cell infusion approaches, whether genetically engineered or not. The expression of the chemokines that mediate the recruitment of NK cells in tumors, i.e., CX3CL1, RARRES2 (Chemerin), CCL5, CXCL9, CXCL10 and CXCL14 [33, 34, 35, 36, 37], was also analyzed, but no major differences were observed among the different tumor indications (Figure S2). The wide majority of solid tumors seem to express NK cell‐attracting chemokines; nevertheless, NK cells represent a minor population of tumor‐infiltrating lymphocytes. Potential explanations include failure to reach the required threshold, physical barriers limiting NK cell infiltration, or a lack of growth factors required to sustain NK cell viability [31, 38, 39].

## 5 Conclusions

Collectively, these data provide an integrative multiomic framework for the rational prioritization of solid tumor indications for NK cell‐based therapies. By systematically combining immunohistochemical evidence of NK cell infiltration, clinical trial efficacy data, transcriptomic profiling, and tumor microenvironment characterization, we identify kidney renal clear cell carcinoma, lung adenocarcinoma and squamous cell carcinoma, cervical and endocervical cancers, and mesothelioma as the tumor types presenting the most favorable biological landscape for NK cell‐mediated antitumor activity. These indications are characterized by meaningful NK cell infiltration and a tumor microenvironment with a favorable balance of activating ligands and limited immunosuppressive signaling.

The clinical trial landscape analysis highlights the current heterogeneity and fragmentation of the NK cell therapy field in solid tumors, with no single intervention or indication yet demonstrating unequivocal superiority. These findings likely reflect the heterogeneity of patients' prior treatment histories and the frequently limited number of evaluable patients in each study. This underscores the urgent need for biomarker‐driven patient selection and standardized endpoints to enable meaningful cross‐study comparisons. Our transcriptomic analyses begin to address this gap by proposing objective, data‐driven criteria for indication selection that extend beyond empirical observation.

Taken together, these findings have direct translational implications. They provide a biologically grounded rationale for focusing NK cell clinical development on a defined set of solid tumor indications, thereby improving the probability of clinical success and accelerating the path toward regulatory approval. As NK cell engineering continues to advance—with increasingly sophisticated CAR constructs, cytokine armoring, and strategies to overcome microenvironmental immunosuppression—the framework presented here offers a roadmap for aligning these innovations with the solid tumor contexts in which they are most likely to be effective. Prospective validation of these findings in dedicated biomarker‐enriched clinical trials will be essential to translate this multimodal evidence into improved patient outcomes.

## Author Contributions

L.C., V.P., T.A.F. and E.V. conceived the presented data. L.C. performed immunohistochemistry data analysis and reviewed clinical trial results. L.C. and Q.B. analyzed and interpreted RNA‐seq data. L.C., V.P., Q.B, T.A.F., and E.V. discussed the results and reviewed the manuscript.

## Conflicts of Interest

The authors declare no conflicts of interest.

## Supporting information




**Supporting File 1**: eji70283‐sup‐0001‐SuppMat.pdf.

**Supporting File 2**: eji70283‐sup‐0002‐Table S1.xlsx.

**Supporting File 3**: eji70283‐sup‐0003‐Table S2.xlsx.

## Data Availability

The data that support the findings of this study are available from the corresponding author upon reasonable request.
