# Supplementary material for: A Multimodal Framework to Guide Solid Tumor Indications for Natural Killer Cell‐Based Therapy
Source: Eur J Immunol. 2026 Sep 16;56(9):e70283. doi: 10.1002/eji.70283 (PMC13579203; doi:10.1002/eji.70283)
Supplement: Supplementary file 1 [file EJI-56-e70283-s003.pdf]

A

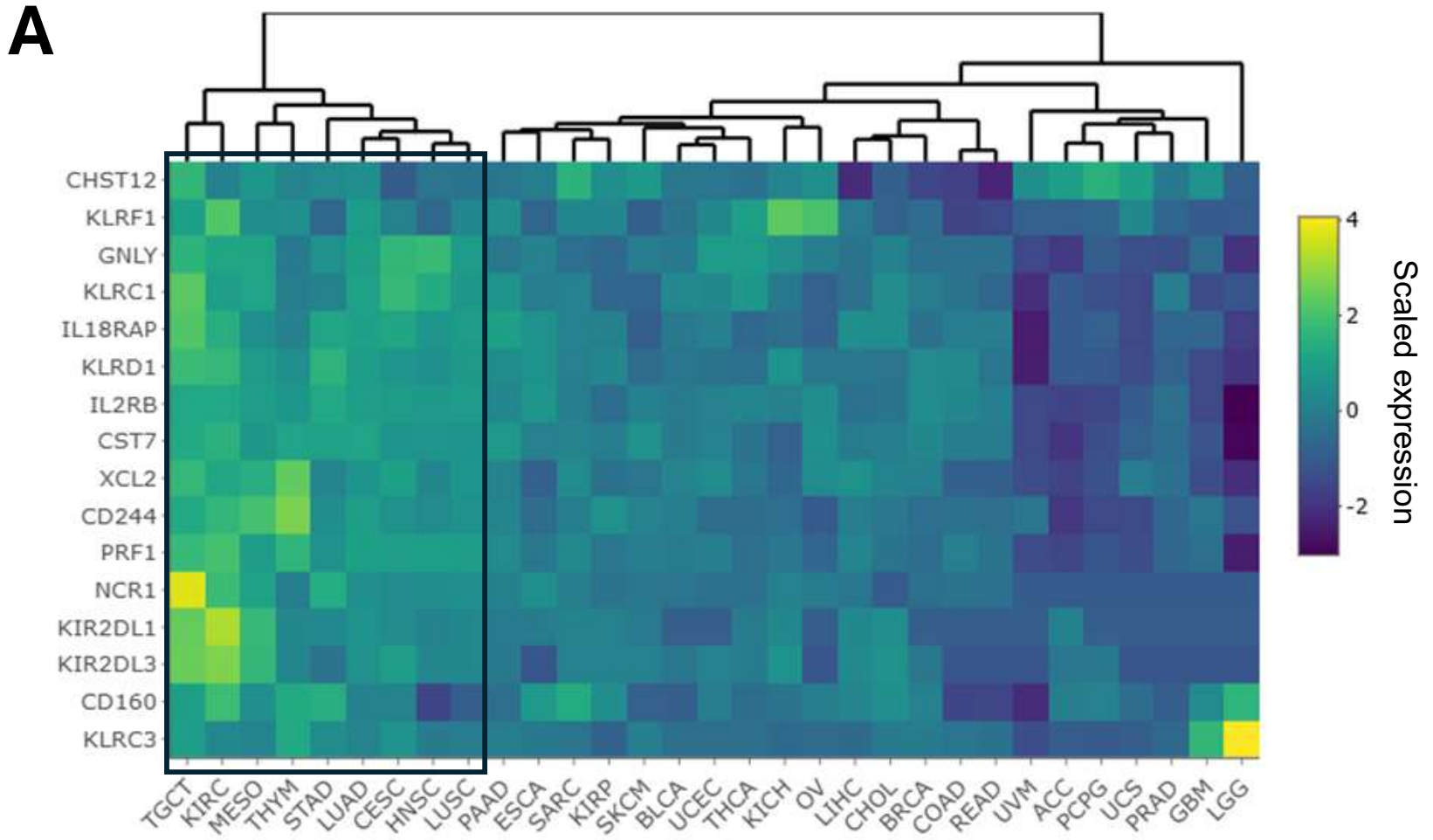

TGCT	Testicular Germ Cell Tumors
KIRC	Kidney rcc
MESO	Mesothelioma
THYM	Thymoma
STAD	Stomach adenocarcinoma
LUAD	Lung adenocarcinoma
CESC	Cervical and endocervical cancers
HNSC	Head and Neck squamous cell carcinoma
LUSC	Lung squamous cell carcinoma

B

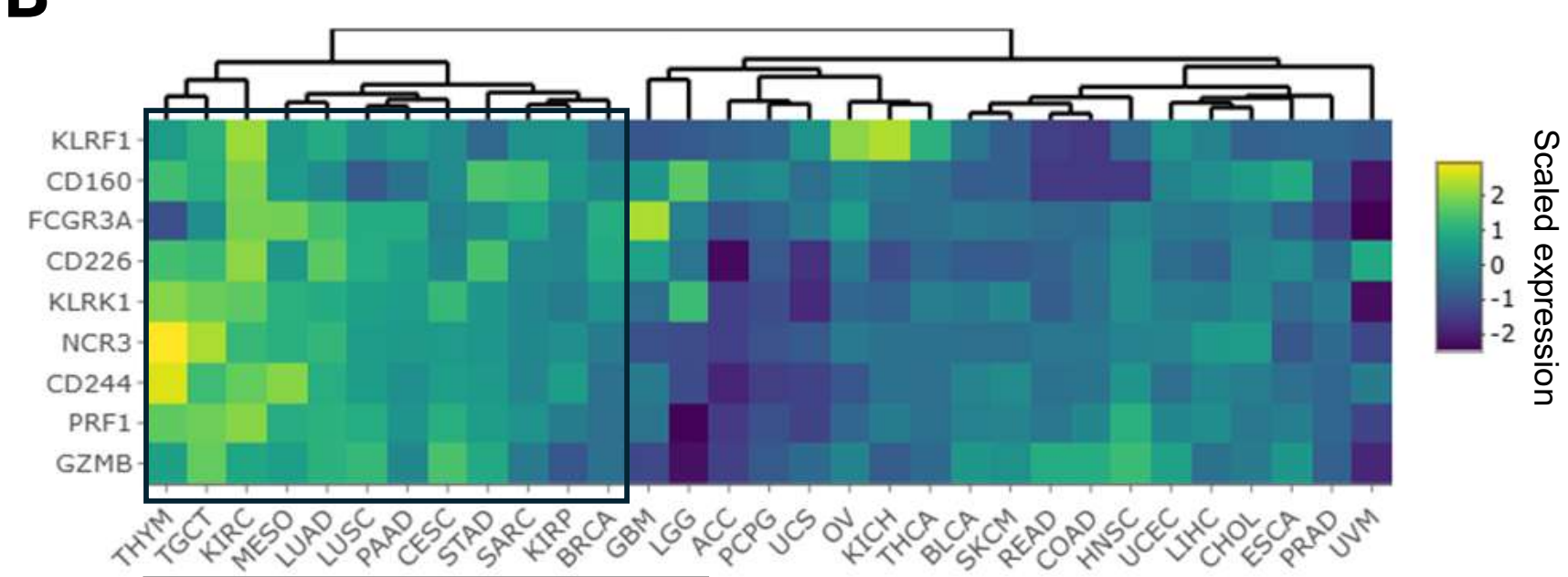

THYM	Thymoma
TGCT	Testicular Germ Cell Tumors
KIRC	Kidney rcc
MESO	Mesothelioma
LUAD	Lung adenocarcinoma
LUSC	Lung squamous cell carcinoma
PAAD	Pancreatic adenocarcinoma
CESC	Cervical and endocervical cancers
STAD	Stomach adenocarcinoma
SARC	Sarcoma
KIRP	Kidney renal papillary cell carcinoma
BRCA	Breast invasive carcinoma

C

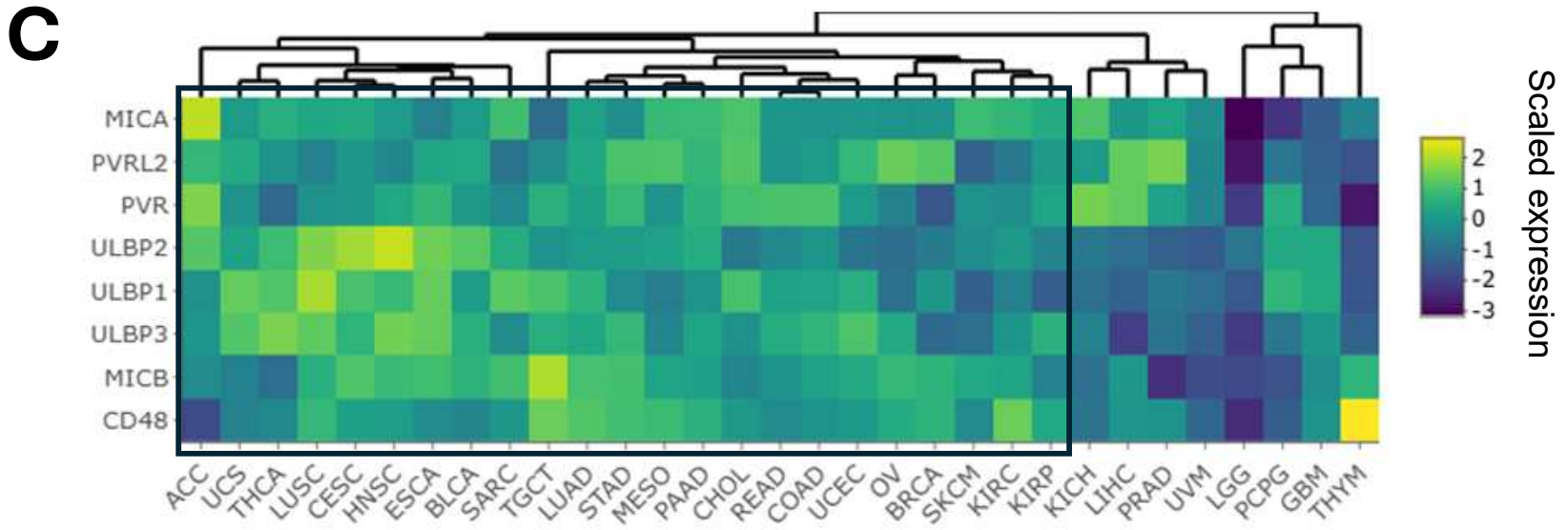

ACC	Adrenocortical carcinoma	MESO	Mesothelioma
UCS	Uterine carcinosarcoma	PAAD	Pancreatic adenocarcinoma
THCA	Thyroid carcinoma	CHOL	Cholangiocarcinoma
LUSC	Lung squamous cell carcinoma	READ	Rectum adenocarcinoma
CESC	Cervical squamous cell carcinoma	COAD	Colon adenocarcinoma
HNSC	Head and Neck squamous cell carcinoma	UCEC	Uterine Corpus Endometrial Carcinoma
ESCA	Esophageal carcinoma	OV	Ovarian serous cystadenocarcinoma
BLCA	Bladder Urothelial carcinoma	BRCA	Breast invasive carcinoma
SARC	Sarcoma	SKCM	Skin Cutaneous Melanoma
TGCT	Testicular Germ Cell Tumors	KIRC	Kidney rcc
LUAD	Lung adenocarcinoma	KIRP	Kidney renal papillary cell carcinoma
STAD	Stomach adenocarcinoma		

D

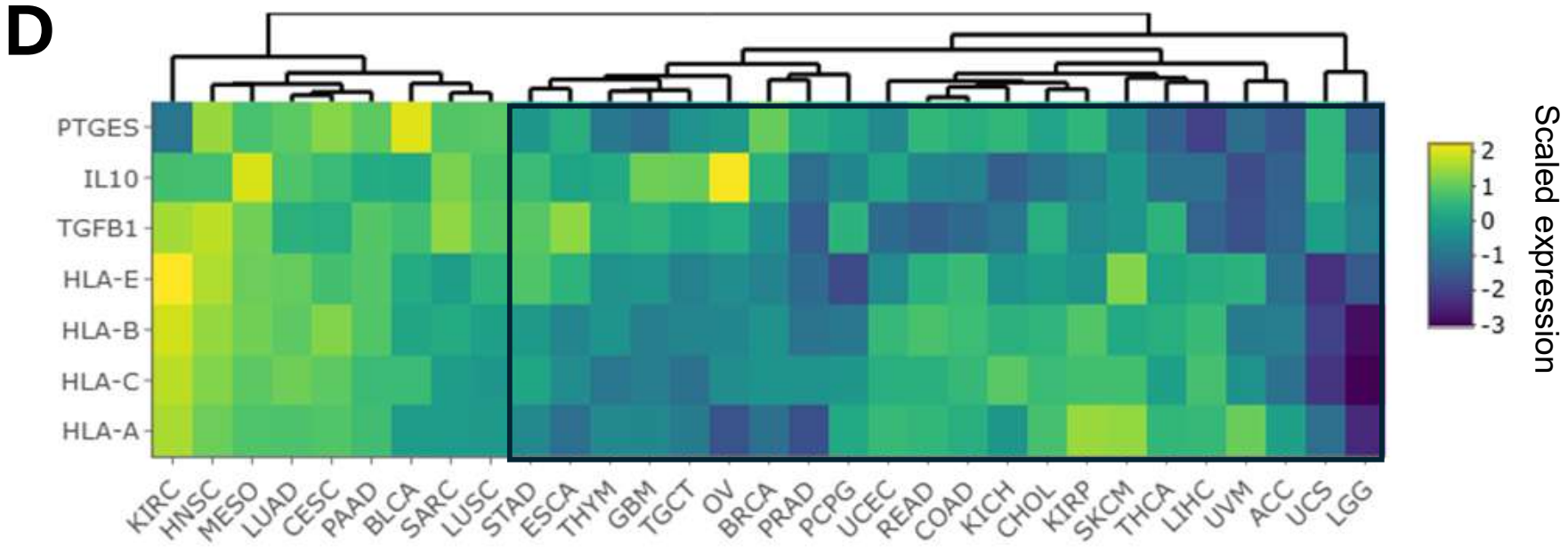

LGG	Brain Lower Grade Glioma
UCS	Uterine Carcinosarcoma
ACC	Adrenocortical carcinoma
UVM	Uveal Melanoma
LIHC	Liver hepatocellular carcinoma
THCA	Thyroid carcinoma
SKCM	Skin Cutaneous Melanoma
KIRP	Kidney renal papillary cell carcinoma
CHOL	Cholangiocarcinoma
KICH	Kidney Chromophobe
COAD	Colon adenocarcinoma
READ	Rectum adenocarcinoma
UCEC	Uterine Corpus Endometrial Carcinoma
PCPG	Pheochromocytoma and Paraganglioma
PRAD	Prostate adenocarcinoma
BRCA	Breast invasive carcinoma
OV	Ovarian serous cystadenocarcinoma
TGCT	Testicular Germ Cell Tumors
GBM	Glioblastoma multiforme
THYM	Thymoma
ESCA	Esophageal carcinoma
STAD	Stomach adenocarcinoma

Supplemental Figure 1

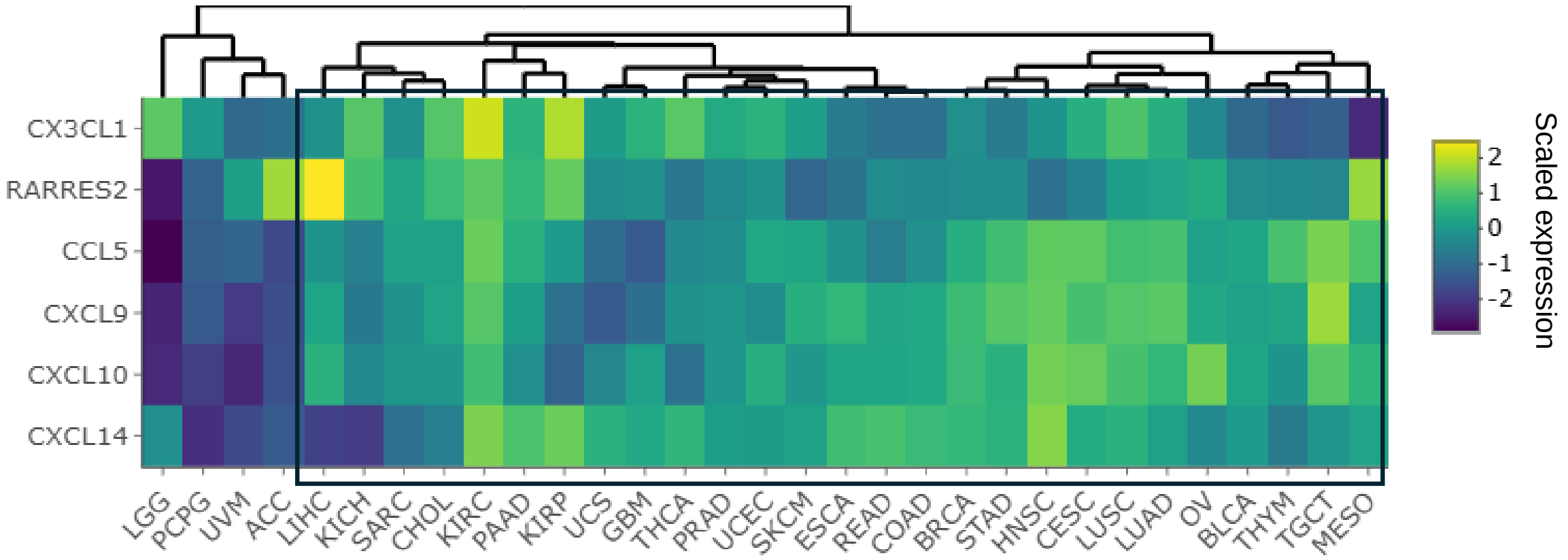

LIHC	Liver hepatocellular carcinoma
KICH	Kidney Chromophobe
SARC	Sarcoma
CHOL	Cholangiocarcinoma
KIRC	Kidney renal clear cell carcinoma
PAAD	Pancreatic adenocarcinoma
KIRP	Kidney renal papillary cell carcinoma
UCS	Uterine Carcinosarcoma
GBM	Glioblastoma multiforme
THCA	Thyroid carcinoma
PRAD	Prostate adenocarcinoma
UCEC	Uterine Corpus Endometrial Carcinoma
SKCM	Skin Cutaneous Melanoma
ESCA	Esophageal carcinoma
READ	Rectum adenocarcinoma
COAD	Colon adenocarcinoma
BRCA	Breast invasive carcinoma
STAD	Stomach adenocarcinoma
HNSC	Head and Neck squamous cell carcinoma
CESC	Cervical and endocervical cancers
LUSC	Lung squamous cell carcinoma
LUAD	Lung adenocarcinoma
OV	Ovarian serous cystadenocarcinoma
BLCA	Bladder Urothelial Carcinoma
THYM	Thymoma
TGCT	Testicular Germ Cell Tumors
MESO	Mesothelioma

Supplemental Figure 2

Supplementary Figure legends

Supplementary Figure 1

Scaled heatmap analysis of RNA-seq datasets from the TCGA database across 31 solid tumor types. **A**, Tumor clustering for the expression of genes indicating the presence of NK cells. **B**, Tumor clustering for the expression of genes coding for activating NK cell receptors and cytotoxic molecules. **C**, Tumor clustering for the expression of the known ligands for activating NK cell receptors. **D**, Tumor clustering for the expression of NK cell inhibitory molecules.

Tumor abbreviations: ACC, Adrenocortical carcinoma; BLCA, Bladder Urothelial Carcinoma; BRCA, Breast invasive carcinoma; CESC, Cervical squamous cell carcinoma and endocervical adenocarcinoma; CHOL, Cholangiocarcinoma; COAD, Colon adenocarcinoma; ESCA, Esophageal carcinoma; GBM, Glioblastoma multiforme; HNSC, Head and Neck squamous cell carcinoma; KICH, Kidney Chromophobe; KIRC, Kidney renal clear cell carcinoma; KIRP, Kidney renal papillary cell carcinoma; LIHC, Liver hepatocellular carcinoma; LUAD, Lung adenocarcinoma; LUSC, Lung squamous cell carcinoma; MESO, Mesothelioma; OV, Ovarian serous cystadenocarcinoma; PAAD, Pancreatic adenocarcinoma; PCPG, Pheochromocytoma and Paraganglioma; PRAD, Prostate adenocarcinoma; READ, Rectum adenocarcinoma; SARC, Sarcoma; SKCM, Skin Cutaneous Melanoma; STAD, Stomach adenocarcinoma; TGCT, Testicular Germ Cell Tumors; THYM, Thymoma; THCA, Thyroid carcinoma; UCS, Uterine Carcinosarcoma; UCEC, Uterine Corpus Endometrial Carcinoma; UVM, Uveal Melanoma. Genes abbreviations: CD160, CD160; CD244, CD244, 2B4; CHST12, Carbohydrate Sulfotransferase 12; CST7, Cystatin F; GNLY, granulysin; IL18RAP, Interleukin 18 receptor accessory protein; IL2RB, CD122; KLRC1, NKG2A; KLRC3, NKG2E; KLRD1, CD94; KLRF1, KLRF1; NCR1, NKp46; PRF1, perforin; XCL2, XCL2; KIR2DL1, KIR2DL1; KIR2DL3, KIR2DL3; CD226, DNAM-1; FCGR3A, CD16; GZMB, granzyme B; KLRK1,

NKG2D; NCR3, NKp30; CD48, CD48; MICA, MICA; MICB, MICB; PVR, PVR; PVRL2, PVRL2; ULBP1, ULBP1; ULBP2, ULBP2; ULBP3, ULBP3; HLA-A, HLA-A; HLA-B, HLA-B; HLA-C, HLA-C; HLA-E, HLA-E; IL10, IL-10; TGFB1, TGF beta 1; PTGES, prostaglandin E2 synthase.

Supplementary Figure 2

The expression of the major NK cell attracting chemokines was analyzed by scaled heatmap analysis of RNA-seq datasets from the TCGA database across 31 solid tumor types.

Tumor abbreviations: ACC, Adrenocortical carcinoma; BLCA, Bladder Urothelial Carcinoma; BRCA, Breast invasive carcinoma; CESC, Cervical squamous cell carcinoma and endocervical adenocarcinoma; CHOL, Cholangiocarcinoma; COAD, Colon adenocarcinoma; ESCA, Esophageal carcinoma; GBM, Glioblastoma multiforme; HNSC, Head and Neck squamous cell carcinoma; KICH, Kidney Chromophobe; KIRC, Kidney renal clear cell carcinoma; KIRP, Kidney renal papillary cell carcinoma; LIHC, Liver hepatocellular carcinoma; LUAD, Lung adenocarcinoma; LUSC, Lung squamous cell carcinoma; MESO, Mesothelioma; OV, Ovarian serous cystadenocarcinoma; PAAD, Pancreatic adenocarcinoma; PCPG, Pheochromocytoma and Paraganglioma; PRAD, Prostate adenocarcinoma; READ, Rectum adenocarcinoma; SARC, Sarcoma; SKCM, Skin Cutaneous Melanoma; STAD, Stomach adenocarcinoma; TGCT, Testicular Germ Cell Tumors; THYM, Thymoma; THCA, Thyroid carcinoma; UCS, Uterine Carcinosarcoma; UCEC, Uterine Corpus Endometrial Carcinoma; UVM, Uveal Melanoma.
